# Blood Pressure, as Affecting Heart, Brain, Kidneys and General Circulation

**Published:** 1905-02

**Authors:** 


Blood Pressure, as Affecting Heart, Brain, Kidneys and General Circulation. By Louis F. Bishop, M.D., Physician to the Lincoln Hospital, New York. One hundred and twelve pages. New York: E. B. Treat & Company. 1904. (Price, $1.00.)

This timely work calls the attention of the profession to the victims of circulatory disease who live from months to years without intelligent treatment, because their physicians have failed to appreciate the early symptoms of disturbance of blood pressure.

The author considers in succession, first, alteration of pressure: second, primary low pressure cases and their management. In this class of patients he lays especial stress upon weakness and strain as an etiological factor. He next considers high pressure cases and their management and especially commends the Nauheim treatment for this class of patients no matter what the origin.

The book closes with a chapter devoted to the estimation of blood pressure and the use of the nitrites for its modification. We commend this book as a thoughtful presentation of the subject. J. W. P.

